# Using florbetapir positron emission tomography to explore cerebrospinal fluid cut points and gray zones in small sample sizes

**DOI:** 10.1016/j.dadm.2015.10.001

**Published:** 2015-11-02

**Authors:** Philip S.J. Weston, Ross W. Paterson, Marc Modat, Ninon Burgos, Manuel J. Cardoso, Nadia Magdalinou, Manja Lehmann, John C. Dickson, Anna Barnes, Jamshed B. Bomanji, Irfan Kayani, David M. Cash, Sebastien Ourselin, Jamie Toombs, Michael P. Lunn, Catherine J. Mummery, Jason D. Warren, Martin N. Rossor, Nick C. Fox, Henrik Zetterberg, Jonathan M. Schott

**Affiliations:** a Dementia Research Centre, Department of Neurodegenerative Disease, UCL Institute of Neurology, London, UK; b Transitional Imaging Group, Centre for Medical Image Computing, University College London, London, UK; c Reta Lila Weston Institute of Neurological Studies, Department of Molecular Neuroscience, UCL Institute of Neurology, London, UK; d Institute of Nuclear Medicine, University College London Hospitals, London, UK; e MRC Centre for Neuromuscular Disease, Department of Molecular Neuroscience, UCL Institute of Neurology, University College London, London, UK; f Clinical Neurochemistry Laboratory, Institute of Neuroscience and Physiology, the Sahlgrenska Academy at the University of Gothenburg, Mölndal, Sweden

**Keywords:** Cerebrospinal fluid, Amyloid PET, Aβ, Tau, Phosphorylated tau, Cut points, Diagnosis

## Abstract

**Introduction:**

We aimed to assess the feasibility of determining Alzheimer's disease cerebrospinal fluid (CSF) cut points in small samples through comparison with amyloid positron emission tomography (PET).

**Methods:**

Twenty-three individuals (19 patients, four controls) had CSF measures of amyloid beta (Aβ)_1–42_ and total tau/Aβ_1–42_ ratio, and florbetapir PET. We compared CSF measures with visual and quantitative (standardized uptake value ratio [SUVR]) PET measures of amyloid.

**Results:**

Seventeen of 23 were amyloid-positive on visual reads, and 14 of 23 at an SUVR of ≥1.1. There was concordance (positive/negative on both measures) in 20 of 23, of whom 19 of 20 were correctly classified at an Aβ_1–42_ of 630 ng/L, and 20 of 20 on tau/Aβ_1–42_ ratio (positive ≥0.88; negative ≤0.34). Three discordant cases had Aβ_1–42_ levels between 403 and 729 ng/L and tau/Aβ_1–42_ ratios of 0.54–0.58.

**Discussion:**

Comparing amyloid PET and CSF biomarkers provides a means of assessing CSF cut points in vivo, and can be applied to small sample sizes. CSF tau/Aβ_1–42_ ratio appears robust at predicting amyloid status, although there are gray zones where there remains diagnostic uncertainty.

## 1 Introduction

Molecular biomarkers are increasingly used to improve diagnostic accuracy in Alzheimer's disease (AD) [1]. In AD, cerebrospinal fluid (CSF) amyloid beta (Aβ)_1–42_ and Aβ_1–42_/Aβ_1–40_ ratio are reduced, and total tau/Aβ_1–42_ ratio and phosphorylated tau (p-tau) both elevated [2]. For these continuous measures to be used diagnostically, dichotomized cut points are often used to define individuals as “AD-positive” or “AD-negative.” Determining such cut points in vivo is not straightforward. It is rarely feasible to seek autopsy confirmation of the presence/absence of AD pathology close to the time of CSF sampling, as has been done for amyloid positron emission tomography (PET) [3], [4]. CSF is typically calibrated against clinical conversion to AD dementia, or in patients versus controls. However, a clinical diagnosis of AD is inaccurate in a proportion of cases [5], and a proportion of apparently healthy elderly individuals have CSF changes consistent with AD [6]. There is considerable between-center variability in CSF assays, and results can be influenced by sample handling [7]. Finally, although dichotomization may be helpful diagnostically, a single cut point is unlikely to be biologically plausible and requires a trade-off between sensitivity and specificity. Perhaps as a result of these factors, CSF biomarker cut points vary widely between centers [8].

Several studies have used data-driven analyses to determine optimal cut points, with one recent large study reporting tau/Aβ_1–42_>0.52 to be the single best discriminator [9], but such approaches are dependent on large sample sizes, usually derived from several centers. How best to determine cut points in individual centers, where recruiting large cohorts is impractical, is less clear. One approach is to compare CSF with other AD molecular biomarkers, such as amyloid PET, now licensed but not yet widely used in routine clinical practice. Visual assessment of F18 florbetapir amyloid PET correlates closely with Aβ deposition at autopsy [3]. Fibrillar Aβ load can be quantified, usually as a standardized uptake value ratio (SUVR) of cortical regions of interest to a reference region (e.g., cerebellum). Previous studies have shown CSF and florbetapir amyloid measures to have similar diagnostic sensitivities [10]. CSF Aβ_1–42_ and florbetapir PET SUVRs correlate closely, particularly at the mid-range of values where cut points are likely to lie [11], [12]; and comparisons between the two have been used to assess potential cut points in a large cohort of patients with mild cognitive impairment (MCI) [13]. We aimed to assess whether comparing CSF and PET biomarkers might provide a means of determining local CSF cut points in relatively small, clinically diverse samples.

## 2 Methods

We recruited 23 individuals: 19 patients with a range of dementia syndromes and four healthy controls. As part of their clinical evaluation, each had a diagnostic lumbar puncture, with CSF samples obtained using a 22G Quincke needle. Optimum CSF handling and transfer procedures were used [14]. Each sample was analyzed for Aβ_1–42_, total tau, and p-tau using INNOTEST enzyme-linked immunosorbent assays (Fujirebio, Ghent, Belgium). Although not in routine clinical use at our center, we also measured Aβ_1–40._ We preferentially chose individuals with CSF Aβ_1–42_ levels in a potential border zone range of 400–700 pg/mL. Each patient's cognition was assessed using the Addenbrook's Cognitive Examination III, scored out of 100 [15].

Each patient had an F18 florbetapir PET scan on a Siemens 3-T PET/MR unit, with a 50-minute dynamic acquisition commencing immediately after intravenous injection of 370 MBq of florbetapir. A volumetric T1-weighted MRI scan was acquired concurrently. Attenuation correction was performed using synthetic computed tomographies (CTs) generated from the MR images [16]. A single static PET image, reconstructed from the last 10 minutes of the PET acquisition, was used for the analysis. PET images were registered to the MRI and segmented using a semiautomated parcellation tool [17].

The four age-matched healthy controls previously had a florbetapir PET/CT scan as part of another study, with a separate T1-weighted MRI acquisition. These images were processed in the same way as described previously, excluding the generation of synthetic CTs. Clinical studies were approved by the Queen Square Research Ethics Committee.

PET images were analyzed in two ways. First, three trained nuclear medicine physicians blinded to the clinical diagnosis visually rated the images positive/negative according to clinical criteria [3]. Second, an SUVR was calculated by comparing uptake in six predefined cortical regions [3] to the whole cerebellum. A positive/negative SUVR cutoff of 1.10 was used as described [18].

Statistical analyses were performed in STATA version 12.0 (College Station, TX, USA). Independent of clinical diagnosis, we compared CSF Aβ_1–42_, Aβ_1–42_/Aβ_1–40_, tau/Aβ_1–42_, and p-tau in subjects rated amyloid positive/negative on visual reads, and based on SUVR. Linear regression was used to assess the relationship between CSF and SUVR, covarying for the interval between lumbar puncture and PET. A secondary analysis compared amnestic and nonamnestic AD clinical syndromes for each of the CSF biomarkers.

## 3 Results

Patients and controls were well matched for age (63.7 ± 7.6 vs. 62.9 ± 7.0). Nine patients had amnestic and 10 nonamnestic (five posterior cortical atrophy, four progressive aphasia, and one behavioral) clinical syndromes (Table 1). CSF examination was done before scanning, with a median delay of 145 days (range, 32–427). Across all subjects, CSF Aβ_1–42_ ranged from 343 to 1199 ng/L, tau/Aβ_1–42_ 0.11–2.54, and p-tau 14–227 g/L.

**Fig. 1:**
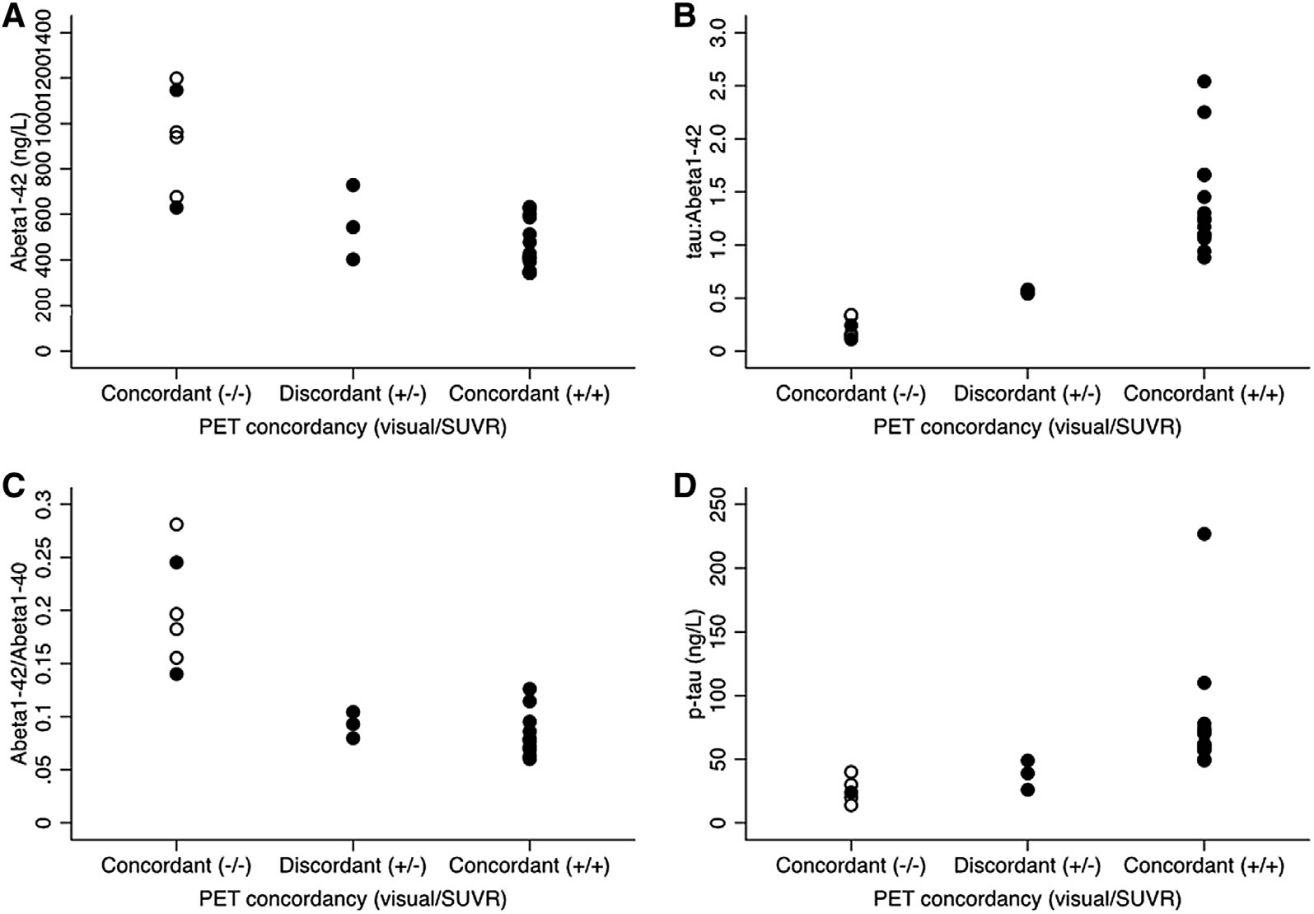
Distribution of CSF values for (A) Aβ_1–42_, (B) tau/Aβ_1–42_, (C) Aβ_1–42_:Aβ_1–40_, and (D) p-tau. Participants have been split, depending on the result of both visual PET read and PET SUVR in to either concordant (−/−) (both PET outcomes negative), discordant (+/−) (one PET outcome positive and one negative), or concordat (+/+) (both PET outcomes positive). Patients are represented by black points, with controls in white. Abbreviations: CSF, cerebrospinal fluid; Aβ, amyloid beta; p-tau, phosphorylated tau; PET, positron emission tomography; SUVR, standardized uptake value ratio.

Seventeen of 23 participants were rated as amyloid-positive on visual assessment. SUVRs ranged from 0.87 to 1.66. At an SUVR cutoff of 1.10, 14 of 23 were amyloid-positive. Comparing SUVR and clinical reads, 20 were concordant (14 positive, six negative); and three discordant (Fig. 1, Table 2). The discordant group all had positive amyloid reads, negative SUVRs (0.88, 1.05, and 1.03), and tau/Aβ ratios between 0.54 and 0.58.

The SUVR correlated with CSF Aβ_1–42_ (R^2^ = 0.26, *P* = .013), CSF Aβ_1–42_/Aβ_1–40_ (R^2^ = 0.32, *P* = .033), CSF tau/Aβ_1–42_ (R^2^ = 0.47, *P* < .001), and CSF p-tau (R^2^ = 0.34, *P* = .005), with no evidence for an influence of duration between CSF sampling and scanning.

At a CSF tau/Aβ_1–42_ ratio cut point of 0.52 [9], the sensitivity and specificity for a positive amyloid scan based on visual reads were 100% (95% confidence interval, 80–100) and 100% (54.1–100), respectively; and based on SUVR, 82% (57–96) and 100% (54–100).

**Fig. 2:**
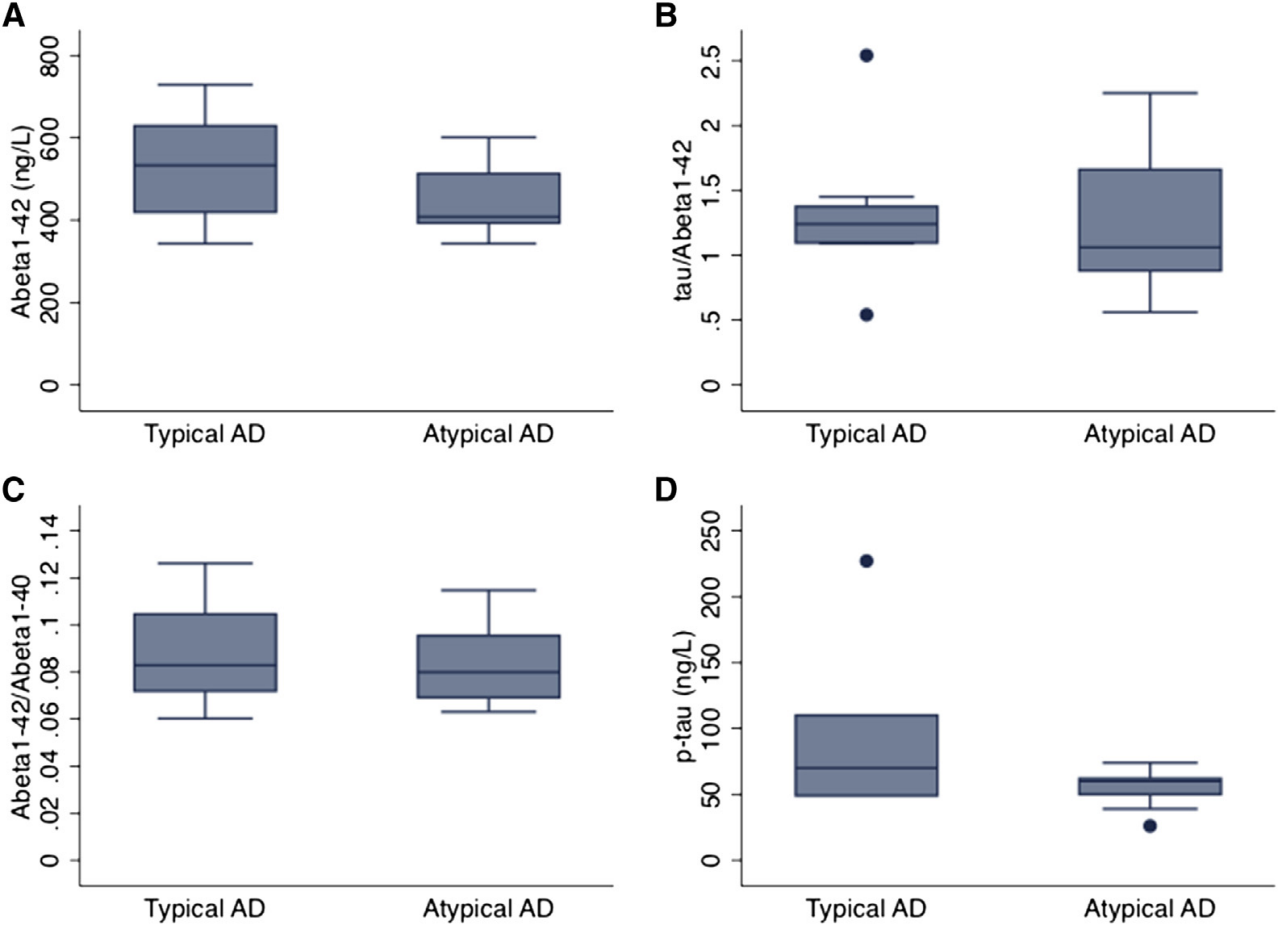
Box plots comparing clinically typical (i.e., amnestic) and clinically atypical cases for (A) CSF Aβ_1–42_, (B) Aβ_1–42_/Aβ_1–40_, (C) tau/Aβ_1–42_, and (D) p-tau. Abbreviations: CSF, cerebrospinal fluid; Aβ, amyloid beta; p-tau, phosphorylated tau; AD, Alzheimer's disease.

Of the patients who were florbetapir positive on visual read (and hence satisfy International Working Group 2 [IWG-2] criteria for AD), there was no significant age difference between the typical (amnestic) AD cases and the atypical (nonamnestic) AD cases (64.3 ± 5.1 vs. 63.8 ± 10.6). When comparing the two subgroups for each CSF biomarker, there were no significant differences, although trend significance was reached for p-tau (*P* = .092), which was higher in typical AD (91.6 ± 63.4 ng/L) compared with atypical AD (55.7 ± 15.3 ng/L; Fig. 2).

## 4 Discussion

As more centers use CSF examination in the investigation of cognitive impairment, local validation of cut points becomes increasingly necessary. Our results show that combining amyloid biomarkers may be a useful means of establishing such cut points. As shown in previous studies [12], [19], there was good correlation between CSF and PET measures of Aβ, noting that we selected individuals in the mid-range of values where linear associations are more likely [11]. When comparing the two methods for determining amyloid positivity, there were some discordant cases—it is not clear whether these reflect misreading by experts, errors in the methodology to calculate the SUVRs, that the SUVR cut point is incorrect [20], or true biological uncertainty. It is notable that in two of the three cases with positive clinical reads but negative SUVR, the latter was close to the cut off of 1.10 (1.03 and 1.05, respectively) and that more consistent relationships between PET amyloid load and CSF were observed using the visual reads.

Considering only individuals with concordant positive/negative PET clinical reads and SUVRs, there was almost complete separation (19 of 20 correctly classified) at a CSF Aβ_1–42_ of 630 ng/L and there was perfect separation on tau/Aβ_1–42_ ratio (positive: ≥0.88, negative: ≤0.34), Aβ_1–42_/Aβ_1–40_ ratio (positive: ≤0.13, negative: ≥0.14 ng/L), and p-tau (positive: ≥49, negative: ≤40 ng/L). A CSF Aβ_1–42_ cutoff of ∼630 ng/L is very similar to those proposed by other recent studies [13], [21] and our results are consistent with a previously determined optimal tau/Aβ_1–42_ cut point [9], supporting the use of this methodology to produce valid cut points in small samples.

Although the concordant cases produced relatively clear cut points, the three discordant cases showed considerable overlap between the positive/negative ranges for Aβ_1–42_ (403–729 ng/L) and p-tau (26–49 ng/L). The tau/Aβ_1-42_ ratios for all three cases were remarkably similar, and in a “gray zone” between 0.54 and 0.58, very close to the previously proposed optimal cut off (0.52) [9]. Although a tau/Aβ_1–42_ ratio of 0.52 had very good sensitivity/specificity for determining amyloid status, these results suggest that rather than a strict dichotomy, introducing a gray zone (e.g., 0.5–0.6) might be more appropriate. However, the significantly narrower gray zone for tau/Aβ_1–42_ than for the other CSF measures assessed is consistent with previous findings that this is likely to be the most robust marker for underlying AD pathology [9], [22]. Aβ_1–42_/Aβ_1–40_ has until now been used less commonly in clinical practice than the other markers but does appear to potentially provide more precise separation of AD-positive and AD-negative cases compared with Aβ_1–42_ alone [23]. However, the overlap in Aβ_1–42_/Aβ_1–40_ values between the PET concordant and PET discordant groups would suggest, consistent with findings from other studies [9], that Aβ_1–42_/Aβ_1–40_ may not be as reliable a marker as tau/Aβ_1–42_.

The gold standard for setting cut points to dichotomize any surrogate biomarker of a continuous biological variable would be to calibrate the cut points against direct measurements of the pathologic entity in question; in this case, brain amyloid and tau. However, the only available method of directly measuring these proteins in the brain is to perform an autopsy. Collecting CSF in a cohort of end-of-life patients to then validate postmortem is not straightforward, and it is unlikely to be feasible to collect sufficient numbers of samples in a relevant time frame in any individual center. In the absence of postmortem pathologic confirmation of diagnosis, we, like a number of other centers, have previously tried to determine individuals with AD or non-AD pathology based on clinical diagnosis, and defined cut points accordingly [9]. However, the clinical diagnosis of AD is known to be unreliable [5], particularly in clinically atypical syndromes, which can be caused by a number of different distinct underlying pathologies [24], [25]. An alternative approach has been to measure CSF degenerative markers in individuals with MCI and then follow-up individuals to find the cut points separating those who do and do not convert to dementia [9]. However, this approach requires large numbers of patients followed over several years making it not feasible for single centers; furthermore, it addresses a related but slightly different clinical questions, i.e., the determination of amyloid-positive individuals versus controls or individuals with non-AD MCI rather than between individuals with AD and non-AD dementias. The unreliability of clinical diagnosis, combined with the variation in assays used, is likely to contribute to the very significant differences between centers with regard to the cut points they use, as exemplified in a recent multicenter study where individual centers' cut points for CSF Aβ_1–42_ varied by over 400 ng/L, from 192 ng/L to 638 ng/L [8]. These differences may also be compounded by the fact that there is no perfect statistical method for determining cut points, which in the absence of a gold-standard with which to compare is inevitably a trade-off between sensitivity and specificity [26]. Unlike CSF measurements, amyloid PET has been validated against postmortem data [3], [4]. Comparing local CSF cut points against a validated in-vivo measure of AD pathology, such as amyloid PET, is, therefore, likely to provide more robust cut points than using clinical diagnoses alone, without the necessity for postmortem examination. The findings of the study have contributed to a change in CSF cut points at our center.

All amyloid-positive individuals in our study had dementia, thus fulfilling IWG-2 criteria for AD [27]. The criteria also allow for patients to be divided according to their clinical presentation in to either typical (amnestic) or atypical (nonamnestic) subgroups. Our study includes a relatively even mixture of typical and atypical cognitive syndromes (Table 1); and in those with biomarker evidence for AD, a mixture of those with IWG-2 typical and atypical AD. When comparing the amnestic and nonamnestic AD cases, there was no evidence of any difference in CSF Aβ or total tau (Fig. 2). There was a suggestion of higher p-tau in typical AD compared with atypical AD, although this only reached trend significance. Although given the small numbers in each of the subgroups, any comparisons should be interpreted with caution; this finding is, however, consistent with previous work performed in larger samples [28]. When assessing all participants together, the p-tau cut point we determined (described previously) is somewhat lower than typically used, perhaps reflecting that a significant proportion of our patients had atypical, nonamnestic presentations.

This study has a number of limitations. The sample size is small, although in keeping with our aim to assess methods for determining cut points in samples appropriate for single centers. There were in some cases significant delays between the CSF and PET scan, although there was no evidence that this influenced the relationship between the two measures; and pragmatically, the fact that delays of some months between CSF and PET do not have a significant influence means that applying this approach in other clinical centers is more feasible. All the patients were scanned on the same PET/MR unit, whereas the controls were scanned on a PET/CT. However, all the controls were very clearly and consistently amyloid negative based on visual read, SUVR, and Aβ_1–42_, suggesting that this is unlikely to have influenced results; and a previous large study has demonstrated excellent concordance between SUVR measurements made in different centers and pipelines [11]. Finally, although amyloid PET correlates well with postmortem pathologic findings, without autopsy confirmation, the true amyloid burden for the individuals in this study is unknown.

## 5 Conclusions

Comparing amyloid PET and CSF biomarkers provides a means of assessing CSF cut points in vivo, and can be applied to small sample sizes. Although in unequivocal cases, a CSF Aβ_1–42_ cut point of ∼630 ng/L and tau/Aβ_1–42_ ratio of ∼0.52 provide good group separation, these data provide evidence that incorporating biomarker gray zones (e.g., 0.5–0.6 for tau/Aβ_1–42_ ratio) may be more biologically plausible.

**Research in context**

1. Systematic review: The authors used PubMed to review the literature pertaining to cerebrospinal fluid (CSF) cut points and, in particular, (1) what methods have been used to determine cut points and (2) what the optimum cut points are thought to be.
2. Interpretation: Our results demonstrate the value of comparing CSF and positron emission tomography biomarkers in relatively small cohorts to assess local CSF cut points. In keeping with previous studies, the tau:amyloid beta_1–42_ ratio was found to be the most robust CSF measure. Although cut points have utility in clinical practice, these data show that in some cases there may be discordance, suggesting the need for biomarker gray zones to reflect diagnostic uncertainty.
3. Future directions: Replication of our approach to determine CSF cut points in other centers will provide further validation. Other future studies should aim to further assess and quantify CSF biomarker gray zones to improve understanding of how best to incorporate these in to clinical practice.

## Figures and Tables

**Table 1:** Clinical details for each of the 19 patients

Patient ID	Age at LP	Clinical presentation	IWG-2 criteria	Coexisting pathology	ACE-III score
1	67.9	PCA	Atypical	No	31
2	59.5	PCA	Atypical	No	67
3	67.6	PPA (logopenic)	Atypical	No	30
4	63.0	Amnestic	—	No	86
5	60.0	PPA (logopenic)	Atypical	No	40
6	61.2	AD (t)	Typical	No	62
7	69.0	PCA	Atypical	Mild SVD	58
8	80.0	Amnestic	Typical	No	86
9	70.9	Frontal	Atypical	No	54
10	57.9	PPA (logopenic)	—	No	93
11	67.0	PCA	Atypical	No	61
12	64.9	Amnestic	Typical	No	86
13	57.9	Amnestic	Typical	No	24
14	59.3	Amnestic	Typical	No	45
15	79.7	PCA	Atypical	Positive VGKC	69
16	56.5	Amnestic	Typical	No	49
17	57.1	Amnestic	Typical	No	31
18	51.7	Amnestic	Typical	No	64
19	58.9	PPA—nonfluent	Atypical	No	70

Abbreviations: ACE-III, Addenbrook's Cognitive Examination III; PCA, posterior cortical atrophy; PPA, primary progressive aphasia; AD, Alzheimer's disease; SVD, small vessel disease; VGKC, voltage-gated potassium channel complex antibodies.

NOTE. For the two patients who were florbetapir negative (on visual read), and so do not satisfy criteria for AD, the IWG-2 column is marked with a−.

**Table 2:** CSF measurements of Aβ_1–42_, tau/Aβ_1–42_, Aβ_1–42_/Aβ_1–40_, and p-tau

	n	Aβ_1–42_ (ng/L) range	Tau/Aβ_1–42_ range	Aβ_1–42_/Aβ_1–40_ range	p-tau (ng/L) range
Positive visual read	17	343–729	0.54–2.54	0.06–0.13	26–227
Negative visual read	6	630–1199	0.11–0.34	0.14–0.28	14–40
SUVR positive	14	343–633	0.88–2.54	0.06–0.13	49–227
SUVR negative	9	403–1199	0.11–0.58	0.08–0.28	14–49
Concordant positive	14	343–633	0.88–2.54	0.06–0.13	49–227
Discordant	3	403–729	0.54–0.58	0.08–0.10	26–49
Concordant negative	6	630–1199	0.11–0.34	0.14–0.28	14–40

Abbreviations: CSF, cerebrospinal fluid; Aβ, amyloid beta; p-tau, phosphorylated tau; PET, positron emission tomography; SUVR, standardized uptake value ratio.

NOTE. Patients are divided based on (1) a positive/negative visual PET read, (2) a positive/negative PET SUVR (using a cutoff of 1.10), and (3) whether there is concordancy or discordancy between the two different PET results.
